# Voluntary Hydration with Skimmed Lactose-Free Milk during Exercise in the Heat: Exploring Effectiveness and Tolerance

**DOI:** 10.3390/nu15092069

**Published:** 2023-04-25

**Authors:** Luis F. Aragón-Vargas, Julián Camilo Garzón-Mosquera, Johnny A. Montoya-Arroyo

**Affiliations:** Human Movement Science Research Center, University of Costa Rica, San José 11501-2060, Costa Rica; julian.garzon@ucr.ac.cr (J.C.G.-M.); jhonny.montoyaarroyo@ucr.ac.cr (J.A.M.-A.)

**Keywords:** exercise, rehydration, GI distress, fluid balance, milk, sports drink, lactose-free skim milk

## Abstract

Replacement of fluid losses (dehydration) during sports activities in the heat has been investigated with different beverages. Bovine milk has been recommended for post-exercise rehydration, but its use during exercise may provoke gastrointestinal disorders. This study compared voluntary fluid intake, hydration, and incidence and severity of gastrointestinal (GI) disorders during exercise in the heat under three conditions: no drink (ND), water (W), and skimmed lactose-free milk (SM). Sixteen physically active university students exercised at 32 °C and 70% RH for 90 min at 60–75% HRmax while drinking W or SM ad libitum, or ND assigned at random. A questionnaire explored possible GI disorders. Ad libitum intake was higher (*p* < 0.05) for water (1206.2 mL) than milk (918.8 mL). Dehydration showed significant differences for SM versus W and ND (W = 0.28% BM; SM = −0.07% BM; ND = 1.38% BM, *p* < 0.05). Urine volume was significantly higher (*p* < 0.05) in the W condition (W = 220.4 mL; SM = 81.3 mL; ND = 86.1 mL). Thick saliva, belching, and abdominal pain were higher for SM, but scores were low. Skimmed lactose-free milk is a suitable, effective alternative to be consumed as a hydration beverage during moderate-intensity cycling in the heat for 90 min.

## 1. Introduction

Physically active humans generate large amounts of heat, some of which is dissipated to the environment by the evaporation of sweat. This sweat loss must be compensated by drinking enough fluid. In an attempt to meet the requirements for effective hydration, several different beverages have been studied. This includes juices [1,2], carbonated drinks [3], carbohydrate-electrolyte drinks [4,5], coconut water [6], and milk [7,8,9,10].

Milk, as a hydration beverage, offers several positive characteristics, such as a concentration of sodium and potassium within those recommended for sports drinks (10–35 mmol/L and 3–5 mmol/L, respectively) [11]. The energy content (90 kcal∙250 mL^−1^ skim milk to 159 kcal∙250 mL^−1^ whole milk) [8] and composition (carbohydrate, protein, and fat) of milk reduce the rate of gastric emptying [12]. A slower gastric emptying rate was originally considered a disadvantage for hydration beverages because of the potential negative gastrointestinal (GI) disorders, and the reduced availability of ingested fluid; on the other hand, it apparently can prevent excessive diuresis [13,14]. Energy from carbohydrates is the preferred source in a hydration beverage and should be between 5 and 10% [11], with milk providing typically 6 to 6.4% without any added sugars.

Meanwhile, milk ingestion in adults has been shown to cause negative GI symptoms, such as fullness and flatulence, often blamed on the presence of lactose [15,16,17]. As an example, Shirreffs, et al. [18] reported a relevant subjective perception of fullness in their subjects up to four hours after ingestion when they drank regular milk post-exercise. These GI problems would recommend against the use of milk for hydration, particularly during exercise. In an attempt to circumvent these problems, researchers have experimented with lactose-free milk [19,20], showing some positive effects. Due to their design, however, these studies prescribe predefined volumes, and the drinks are ingested at rest after exercise. There remain multiple questions regarding the effectiveness of lactose-free skim milk as a hydration beverage: do exercising subjects drink enough milk ad libitum? How does the GI tract respond to this ingestion during exercise?

The purpose of this study was, therefore, to compare voluntary fluid intake with water or lactose-free skim milk during moderate-intensity exercise in the heat, and to compare the resulting urine output and hydration effectiveness. The study also explored GI distress, comparing 19 upper tract, lower tract, and systemic GI symptoms under three conditions: water, milk, and no drink.

## 2. Materials and Methods

16 male and female university students between 18 and 35 years old were evaluated. They were apparently healthy, per the Physical Activity Readiness Questionnaire [21], and physically active according to the American College of Sports Medicine criteria [22]. Specific questions aimed to screen for cardiovascular, renal, or hepatic problems, and to verify that no medication was being used during the study. Participants were requested to abstain from caffeinated and alcoholic beverages, from diuretics and stimulants, and from training hard on the 24 h prior to each visit to the laboratory. Informed consent was obtained from all subjects involved in the study; the protocol was approved by the University of Costa Rica Institutional Review Board, CEC-517-2019.

Participants came to the laboratory after fasting for ten hours or more. A urine sample was obtained to verify urine specific gravity (USG) with a manual refractometer (ATAGO^®^, model URC–Ne, d 1.000–1.050, Minato-ku, Tokyo, Japan); euhydration was accepted if USG ≤ 1.020. Each participant ingested a standard breakfast: 1573 kJ (376 kcal), 11% fat, 14.5% protein, and 74% carbohydrate, including 200 mL of fluids and approximately 876 mg sodium. Thirty minutes later, they were weighed nude and dry to the nearest 10 g on a calibrated scale (e-Accura^®^, model DSB291, Qingpu, Shanghai, China); this body mass was used as the pre-exercise value (BMpre). Baseline gastrointestinal (GI) symptoms were evaluated with a questionnaire adapted from Pfeiffer, et al. [23], a Likert scale ranging from 0 (no problem) to 9 (the worst it has ever been).

All participants exercised under all three conditions, in random order, a minimum of two days apart: no drink (ND), drinking water ad libitum (W), or drinking lactose-free, skimmed milk ad libitum (372 kJ—89 kcal, 8 g protein, 0.5 g fat, and 98 mg sodium in 250 mL) (SM). They pedaled a stationary bike (Schwinn AC Performance Plus, Vancouver, WA, USA) at 60 to 75% HRmax for 90 min. Maximum heart rate (Hrmax) was calculated according to Tanaka, et al. [24]; exercise intensity was monitored with a Polar^®^ heart rate monitor (Model FT7, Kempele, Finland) and perceived exertion reported using Borg’s 6–20 scale [25] every 15 min. Participants exercised in the heat (32 °C dry bulb, 70% relative humidity) in a controlled environment chamber; environmental heat stress was monitored with a 3M device (Questemp36^®^, Oconomowoc, WI, USA). Beverages were maintained at 12 °C with a PolyScience^®^ circulation bath (Model MX20R-30-A11B, Niles, IL, USA); fresh bottles were delivered every 15 min. Each bottle was weighed to the nearest gram before and after drinking with a digital OHAUS^®^ compact scale (model CS2000, Parsippany, NJ, USA); voluntary fluid intake from each bottle was calculated as the difference in grams, assuming 1 g = 1 mL; voluntary intake from all bottles was added up for each participant for each condition. Each exercise session was divided into three 30 min periods followed by a 5 min rest to complete the GI symptoms questionnaire and to measure nude and dry body mass.

Dehydration was calculated from body mass loss during the exercise period as follows:

%DEHY = [(BM_90_ − BM_0_)/BM_0_] × 100 (1)

Sweat rate was calculated from body mass loss in grams during the exercise period, as well as urine output and fluid intake in mL, according to the formula:

Sweat rate = (BM_0_ − BM_90_ + Fluid intake − Urine output)/1.5 h (2)

Net Fluid Balance was calculated for each time point, using body mass immediately before exercise as the reference, as follows:

NFB_time_ = BM_time_ − BM_0_ (3)

This is a randomized, cross-sectional study. Descriptive statistics (mean, standard deviation, and range) were calculated for age, height, and weight to characterize the sample. All variables were checked for normality. To verify that circumstances were the same for all conditions, one-way ANOVAs were performed for the baseline measurements (BM, USG) and for the average exercise intensity (HR, RPE), dry bulb temperature, and relative humidity during the exercise sessions. Dependent variables were analyzed as follows: voluntary fluid intake was compared only between milk and water with a one-way ANOVA. Urine output (UO) and dehydration (%DEHY) were compared among all three conditions with one-way ANOVAs. Net Fluid Balance (NFB) was analyzed first with a two-way, repeated-measures ANOVA to explore the relevance of both quadratic and linear functions of the variables of interest. After verification, a regression model with the subjects included as blocks; and time and condition, as well as their interaction as potential predictors, was evaluated. NFB was the dependent variable.

GI distress was analyzed in two different ways. First, scores for each symptom were analyzed with a two-way, repeated-measures ANOVA (3 conditions × 4 time points: 0, 30, 60, and 90 min post-exercise). In addition, symptom scores were transformed to nominal data, assigning a 0 for “absent” and a 1 for “present”, regardless of the time or the intensity, for each participant and each condition. This information was used to perform a logistic regression on each of the 19 symptoms. All analyses were performed with JMP^®^ Pro 15 (SAS Institute, Inc., Cary, NC, USA).

## 3. Results

16 participants (seven females, nine males) completed testing for the three conditions. Their basic characteristics (mean ± S.D.; range): age = 24.3 ± 4.6; 18–34 years old. Height = 164.8 ± 6.9; 151–175 cm. Body weight = 67.4 ± 15.1.; 49.1–105.2 kg. A post hoc statistical power analysis was performed to confirm that a sample of 16 participants was adequate for power > 0.85, with α < 0.05 and a specific, meaningful mean difference for each variable (see Table 1).

One-way analyses of variance showed that W, SM, and ND trials were performed under similar ambient temperature (*p* = 0.2253) and relative humidity (*p* = 0.3919), with a similar exercise intensity (*p* = 0.8504 for average heart rate and *p* = 0.8314 for RPE), and with similar sweat rates (*p* = 0.5099). In addition, participants arrived with a similar hydration status (*p* = 0.8915 for initial USG and *p* = 0.8806 for baseline body mass) (see Table 2).

Table 2 also shows mean values for the variables of interest. Voluntary fluid intake was higher for W than for SM (mean difference; 95% C.I.): 287.4 mL; 18.9 to 555.9 mL. Urine output was higher for W than for SM (139.1 mL; 47.5 to 230.7 mL) and higher for W than for ND (134.3 mL; 42.7 to 225.9 mL); SM and ND were not different (*p* > 0.05). Dehydration was higher for ND than SM (1.48% BM; 0.87 to 2.02% BM) and higher for ND than W (1.10% BM; 0.53 to 1.67% BM), with no difference between W and SM (*p* > 0.05).

The ANOVA on Net Fluid Balance showed that a quadratic function of time was not significant (*p* = 0.1232), nor was its interaction with condition (*p* = 0.9389). There was, however, a time-by-condition interaction (*p* < 0.0001), as well as a significant effect of subject (*p* = 0.0003). This model was statistically significant (*p* < 0.0001) with an R-squared = 0.65. Therefore, the linear regression model used included subjects (as blocks), measurement time, condition, and the time-by-condition interaction (see Table 3).

**Figure 1:**
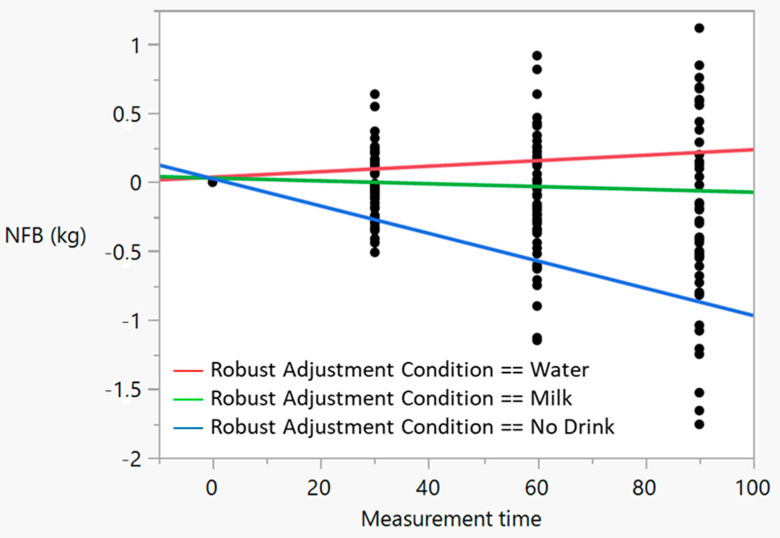
Bivariate adjustment of NFB as a function of measurement time. Robust estimators were 0.002 for water (*p* = 0.1875), −0.001 for milk (*p* = 0.4035), and −0.01 for ND (*p* < 0.0001).

Figure 1 shows the tendency of NFB over time for each condition; because of heteroscedasticity, robust adjustments were calculated for the three conditions. In the no drink condition, the robust estimator was −0.01; that is, for every 30 min, NFB decreased 300 g (*p* < 0.0001). Robust estimators for W and SM were not statistically different from zero (*p* > 0.05).

GI distress was totally absent for several symptoms, namely vomiting, nausea, loose stools/diarrhea, and headache, precluding any comparisons. Of the remaining 15 symptoms, five showed significant differences among conditions (see Table 4). Abdominal distension was greater for ND than for W (0.22; 0.004 to 0.43) (mean difference; 95% C.I.). Thirst was greater for ND than for W (0.31; 0.05 to 0.58), and greater for ND than for SM (0.31; 0.05 to 0.58). Abdominal pain was greater for SM than for W (0.44; 0.14 to 0.74), and greater for SM than for ND (0.41; 0.11 to 0.71). Thick saliva was greater for SM than for W (0.48; 0.19 to 0.78), and greater for SM than for ND (0.31; 0.01 to 0.61). Finally, belching was greater for SM than for W (0.61; 0.21 to 1.01), and greater for SM than for ND (0.67; 0.27 to 1.07).

Logistic regression on each of the GI symptoms was undefined for twelve symptoms, because there were too many zeroes. Non-significant odds ratios (O.R.) were obtained for abdominal distension (*p* ≥ 0.1939), flatulence (*p* ≥ 0.2017), abdominal pain (*p* ≥ 0.2388), dizziness (*p* ≥ 0.4019), urge to urinate (*p* ≥ 0.3233), and thick saliva (*p* ≥ 0.4166). Only belching showed a significant O.R. = 27.99 (*p* = 0.0231) for milk vs. water, meaning that the odds of reporting belching were 28 times larger when drinking water than when drinking milk (lower limit of the 95% C.I. = 1.58).

## 4. Discussion

The most important finding of this exploratory study was that voluntary fluid intake with lactose-free skim milk was enough to maintain euhydration during 90 min of cycling exercise at 60–75% HRmax (mean RPE = 11.8) in the heat (32 °C, 70% RH). Net Fluid Balance was maintained both with milk and with water intake, while heat stress was enough to reduce NFB by 0.937 kg in the ND condition. Similarly, dehydration was statistically not different from zero for both SM and W (Table 1), while it reached an average of 1.38% BM by the end of the exercise session in the no drink condition. Voluntary fluid intake was higher with water than milk, compensating for the higher urine output in the W condition and resulting in no statistical difference in NFB between the drinks.

Voluntary fluid intake is influenced by a number of physiological, environmental, and psychological factors, including fluid palatability and beverage temperature [26]. In this study, all drinks were delivered to participants at 12 °C, although it is possible that ingestion took place closer to the ACSM-recommended range of 15–22 °C [27], as the beverages warmed up in the room before consumption. Nevertheless, there was no difference in beverage temperature between SM and W. We did not evaluate palatability; participants may have ingested more water than milk because of habit or because they found it more palatable in the study conditions. It is also possible that voluntary intake was influenced by some adverse GI symptoms experienced with SM. This is discussed below.

Urine output has been shown to be higher when drinking water [28] and lower when drinking milk [29] when fluids are ingested during exercise in the heat. This has been attributed to the electrolyte content of milk [13]; the results of the present study confirm previous findings. The inability of the renal system to retain enough fluids in response to active rehydration, during or after exercise in the heat, is called the renal paradox; urine output might be expected to be null while the person is dehydrated, but it tends to be considerably higher [30]. In the present study, urine output was low (median = 102.5 mL in 90 min for the drinking conditions), allowing euhydration to be maintained during exercise in the heat. Longer follow-up post-exercise may show different results.

Under this particular protocol, voluntary fluid intake was enough to maintain euhydration during exercise. Previous studies have shown conflicting results. In a simulated 16 km outdoor race at 20.5 °C and 64% RH, Passe, et al. [31] tested voluntary fluid intake in 18 seasoned marathoners (15 males, 3 females). They recorded an average dehydration of 1.9 ± 0.8% BM, despite having an ample supply of a cold sports drink. On the other hand, Solera-Herrera and Aragón-Vargas [32] reported a negligible average dehydration of 0.14 ± 0.98% BM (range: 2.3% to −2.85% BM) in 94 college students who alternated simulated skiing, stationary cycling, and bench-stepping (a moderate-intensity exercise of 60% HRreserve) for one hour in a controlled-environment laboratory at 30 °C and 70% RH. They also had an ample supply of cold sports drink. Passe, et al. [31] acknowledge that because of the competitive nature of their exercise task, it is possible that their subjects intentionally restricted their fluid intake (6.1 ± 3.4 mL × kg^−1^ × h^−1^, compared with 17.3 ± 9.3 mL × kg^−1^ × h^−1^ [32]). In the present study, voluntary intake normalized by body mass was 12.0 ± 5.5 mL × kg^−1^ × h^−1^ with water and 9.3 ± 5.8 mL × kg^−1^ × h^−1^ with milk, but overall sweat rate was moderate (median = 483.7 mL/h). This additional voluntary consumption of water is important because water causes a higher urine output, to the point that humans are unable to maintain euhydration after exercise in the heat when post-exercise rehydration with water is prescribed at 150% of sweat loss [18].

This exploratory study evaluated a wide variety of GI symptoms which were expected to be lower than previously reported for various drinks, because of the utilization of lactose-free, skimmed milk. Lactose intake causes symptoms of bloating, flatulence, abdominal pain, and diarrhea in a large segment of the population [33]. In addition, this type of milk was expected to be isotonic (270–330 mOsm/kg H_2_O isotonic beverage range [34]), with an osmolality somewhere between that reported for low-fat bovine milk but lower than reported for lactose-reduced cow’s milk (273 and 389 mOsm/kg H_2_O, respectively) [35]; in fact, it turned out to be slightly hypertonic at 438 mOsm/kg H_2_O. Hypertonic drinks have been associated with greater GI disturbances [36,37]. Despite its higher osmolality, the sodium concentration of 17 mEq/L and very low-fat content (2 g/L), are similar to an optimal rehydration beverage [11].

Under the study conditions, namely, 90 min of pedaling at moderate to high intensity in humid heat while drinking water or milk ad libitum, or ND, there were no reports of vomiting, nausea, loose stools/diarrhea, or headache. A majority of symptoms were very mild and not different among conditions (see Table 4); fullness and urge to urinate scored the highest, but even for them, the group means at specific points in time did not exceed 1.19 on a scale from 0 to 9. This compares favorably to the reports of Pfeiffer, et al. [38], where the participants reported ingesting similar volumes (mean = 643 mL/h) but were asked about their experience during sports competitions that lasted for over two hours. Even though their participants reported fewer GI problems during cycling than while running or competing in triathlons, 4% of the cyclists reported serious symptoms. Peters, et al. [39] also reported higher GI symptom prevalence than the present study, but they did not report symptom intensity. Their subjects were also involved in high-intensity, 3 h running and cycling exercise. It is likely that the milder symptoms in our study resulted from a shorter-duration, moderate-intensity cycling exercise.

The exercise protocol in the present study was enough, however, to produce significant differences among conditions in five GI symptoms (Table 4). Similar to those mentioned in the previous paragraph, scores for these five symptoms were low. Thick saliva was found to be greater for milk than for the other two conditions. Salivary viscosity has been shown to increase during exercise [40], apparently the result of increased secretion of salivary mucin MUC5B; the latter mechanism has not been studied in association with drinking. It is possible that the protein content in milk may increase saliva viscosity to a greater extent than the documented increase with exercise alone; this “mouth-coating” swallowing difficulty effect has been documented at rest [41]. Mean values reported in the present study for thick saliva in the milk condition did not exceed 1.0, suggesting that the phenomenon is perceptible but not problematic.

Both abdominal pain (mean ≤ 0.63) and belching (mean ≤ 1.19) scores were low but significantly higher for milk than for the other two conditions. It might be speculated that this obeys the slower gastric emptying and intestinal absorption of milk, apparent in the study by Shirreffs, Watson, and Maughan [13], which has been reported to cause a greater sensation of fullness [29]. In our study, however, fullness (mean ≤ 1.19) was not different among conditions, and abdominal distension (mean ≤ 0.56), a similar symptom, was actually greater for ND than for W (and not different for milk). In addition, our logistic regression did not confirm our own analysis of the mean scores, as only belching showed a significant odds ratio, and that in the opposite direction of our expectations (the odds of reporting belching were higher when drinking water than when drinking milk). Belching is frequently reported during exercise [42,43,44], and it is attributed to oral ingestion of excessive amounts of air [45], which in the present study may have occurred concomitantly with drinking a large volume of water [46]. Resolution of the discrepancies mentioned above regarding fullness, abdominal distension, abdominal pain, and belching may require different protocols; perhaps a more demanding exercise duration and intensity, the explicit prescription of higher fluid volumes, or a combination of both would result in higher symptom scores and frequencies which might provide clearer results.

Finally, we evaluated thirst, which is not really a GI symptom but is a very relevant perception during exercise. Thirst was higher when the subjects were not allowed to drink than when they drank water or milk ad libitum. This result conforms to logic, but also to experiments where thirst has been shown to be a good indicator of fluid needs in the absence of drinking [47].

An important comment must be made regarding the exercise protocol. In this study, the exercise mode and intensity were selected as a means to induce dehydration; they should be the same for all three conditions. Intensity is hereby reported in terms of heart rate and ratings of perceived exertion, but no absolute measure of workload is provided. This should not be a problem as far as individual differences go; while not all participants might have exercised at the same relative intensity, the important issue is that the intensity was not different among conditions, as can be verified in Table 2. Heart rate remained constant over time: a 3 (condition) × 6 (time) ANOVA showed no interaction (F = 1.35, *p* = 0.26) and no main effects of time (F = 1.70, *p* = 0.19) or condition (F = 0.81, *p* = 0.45). On the other hand, it is true that cardiovascular drift (not measured) may have been higher during the ND trial because of higher dehydration, resulting in a lower absolute workload for each participant in that condition. While ND did not show a different sweat rate, suggesting a similar workload, the fact that a constant workload was not prescribed for each participant is a limitation of the present study.

## 5. Conclusions

Voluntary drinking of skimmed lactose-free milk or plain water was sufficient to maintain euhydration during 90 min of moderate-intensity exercise in the heat. Milk was more efficient, requiring a smaller volume than water, thanks to lower urine output. A few GI disturbances were higher when drinking milk: thick saliva, belching, and abdominal pain, but their average scores were low, and the only significant Odds Ratio, belching, was actually favorable to milk vs. water. Lactose-free skim milk is hereby documented to be a suitable, effective hydration beverage for 90 min of moderate cycling in the heat. A more demanding exercise, requiring higher fluid intake, may give different results.

## Figures and Tables

**Table 1:** Statistical power for variables of interest, n = 16, α < 0.05. (*) Fullness required a meaningful mean difference of 0.26 a.u. to meet this statistical power; urge to urinate required 0.45 a.u.

Variable	Meaningful Mean Difference	Statistical Power
Final urine specific gravity	0.003	0.9345
Voluntary fluid intake	220 mL	0.9036
Urine volume	60 mL	0.9302
Percent dehydration	0.5% BM	0.9964
Sweat rate	75 mL/h	0.8606
GI symptoms	0.2 a.u.	>0.85 *

**Table 2:** Physiological and environmental variables by condition (n = 48).

Variable	Water (Mean)	Milk (Mean)	No Drink (Mean)
Room temperature (°C)	32.0	32.1	31.7
Room relative humidity (%)	69.3	69.0	70.1
Average heart rate (bpm)	134.9	136.3	135.9
Average rating of perceived exertion	11.8	11.8	11.7
Baseline body mass (kg)	67.41	67.36	67.43
Initial Urine Specific Gravity	1.018	1.018	1.019
Final Urine Specific Gravity	1.016 ^a^	1.022	1.021
Voluntary fluid intake (mL)	1206.2 ^b^	918.8	0 ^d^
Urine output (mL)	220.4 ^a^	81.3	86.1
Dehydration (% BM)	0.28	−0.07	1.38 ^c^
Sweat rate (mL/h)	538.5	599.6	567.2

^a^ Different from SM and ND (*p* < 0.05). ^b^ Different from SM (*p* = 0.0375). ^c^ Different from SM and W. (*p* < 0.05). ^d^ Not compared; by design, this was not a variable.

**Table 3:** Net fluid balance effects test.

Effects Tests	Sum of Squares	F-Value	Probability > F
Condition	0.004	0.029	0.971
Measuring time	1.907	25.725	<0.0001
Condition × measurement time	6.294	42.437	<0.0001
Subject of measurement	3.507	2.956	0.0002

**Table 4:** Ratings for GI symptoms over time. Scale from 0 (not at all) to 9 (the worst I have felt in my life).

Symptom	0 min			30 min			60 min			90 min			
	W	SM	ND	W	SM	ND	W	SM	ND	W	SM	ND	*p*-Value (Cond.)
Reflux	0	0	0.25	0	0.44	0.25	0	0.44	0.25	0	0.44	0.25	0.0685
Heartburn	0	0.38	0	0	0.13	0	0	0	0	0	0	0	0.1873
Ab. distension	0	0	0.25	0	0.20	0.56	0.06	0.13	0.19	0.06	0.06	0	0.0492 ^a^
Cramping	0	0	0	0	0	0	0	0.19	0	0	0	0	0.3701
Vomiting	0	0	0	0	0	0	0	0	0	0	0	0	------
Nausea	0	0	0	0	0	0	0	0	0	0	0	0	------
Intestinal cramps	0	0	0	0	0	0	0	0	0	0	0.25	0	0.3701
Urge to defecate	0	0	0	0	0	0	0	0.19	0	0	0	0	0.3701
Flatulence	0	0.19	0	0.31	0.31	0.06	0.25	0.56	0.06	0.25	0.25	0	0.0635
Abdominal pain	0	0	0	0	0.63	0.13	0	0.63	0	0	0.50	0	0.0008 ^b^
Loose stools/diarrhea	0	0	0	0	0	0	0	0	0	0	0	0	------
Dizziness	0	0	0	0	0	0.13	0.13	0	0.06	0.19	0.13	0.13	0.1439
Headache	0	0	0	0	0	0	0	0	0	0	0	0	------
Muscle cramping	0	0	0	0.19	0.25	0.44	0.06	0.13	0.38	0	0.06	0.38	0.0972
Urge to urinate	0	0	0	0.88	0.13	0.88	1.38	0.75	1.19	1.19	0.38	0.63	0.2464
Thick Saliva	0	0	0	0	0.31	0	0	0.69	0.31	0	0.94	0.38	0.0007 ^b^
Belching	0	0	0	0.19	1.19	0	0.31	1.00	0.19	0.25	1.00	0.31	0.0001 ^b^
Fullness	0.31	0.31	0.31	0.31	0.69	0.31	0.63	1.19	0.63	0.56	0.94	0.50	0.0861
Thirst	0	0	0	0	0	0.38	0	0	0.44	0	0	0.44	0.0283 ^a^

(W) Water. (SM) Milk. (ND) No Drink. ^a^ Difference between no drink vs. water and milk. ^b^ Difference between milk vs. water and no drink.

## Data Availability

Publicly available datasets were analyzed in this study. This data can be found here: https://hdl.handle.net/10669/88256 (accessed on 10 March 2023).
